# Older Veterans’ Experiences of a Multicomponent Telehealth Program: Qualitative Program Evaluation Study

**DOI:** 10.2196/46081

**Published:** 2023-09-08

**Authors:** Michelle R Rauzi, Meredith L Mealer, Lauren M Abbate, Jennifer E Stevens-Lapsley, Kathryn A Nearing

**Affiliations:** 1 Physical Therapy Program Department of Physical Medicine and Rehabilitation University of Colorado Aurora, CO United States; 2 Department of Physical Medicine and Rehabilitation School of Medicine University of Colorado Aurora, CO United States; 3 Mental Illness Research Education and Clinical Center Veterans Affairs Eastern Colorado Healthcare System Aurora, CO United States; 4 Geriatric Research Education and Clinical Center Veterans Affairs Eastern Colorado Healthcare System Aurora, CO United States; 5 Division of Geriatric Medicine School of Medicine University of Colorado Aurora, CO United States

**Keywords:** telehealth, multimorbidity, older adults, veteran health, physical therapy

## Abstract

**Background:**

There are 8.8 million American veterans aged >65 years. Older veterans often have multiple health conditions that increase their risk of social isolation and loneliness, disability, adverse health events (eg, hospitalization and death), mental illness, and heavy health care use. This population also exhibits low levels of physical function and daily physical activity, which are factors that can negatively influence health. Importantly, these are modifiable risk factors that are amenable to physical therapy intervention. We used a working model based on the dynamic biopsychosocial framework and social cognitive theory to conceptualize the multifactorial needs of older veterans with multiple health conditions and develop a novel, 4-component telehealth program to address their complex needs.

**Objective:**

This study aims to describe veterans’ experiences of a multicomponent telehealth program and identify opportunities for quality and process improvement. We conducted qualitative interviews with telehealth program participants to collect their feedback on this novel program; explore their experience of program components; and document perceived outcomes and the impact on their daily life, relationships, and quality of life.

**Methods:**

As part of a multimethod program evaluation, semistructured interviews were conducted with key informants who completed ≥8 weeks of the 12-week multicomponent telehealth program for veterans aged ≥50 years with at least 3 medical comorbidities. Interviews were audio recorded and transcribed. Data were analyzed by a team of 2 coders using a directed content analysis approach and Dedoose software was used to assist with data analysis.

**Results:**

Of the 21 individuals enrolled in the program, 15 (71%) met the inclusion criteria for interviews. All 15 individuals completed 1-hour interviews. A total of 6 main conceptual domains were identified: technology, social networks, therapeutic relationship, patient attributes, access, and feasibility. Themes associated with each domain detail participant experiences of the telehealth program. Key informants also provided feedback related to different components of the program, leading to adaptations for the biobehavioral intervention, group sessions (transition from individual to group sessions and group session dynamics), and technology supports.

**Conclusions:**

Findings from this program evaluation identified quality and process improvements, which were made before rigorously testing the intervention in a larger population through a randomized controlled trial. The findings may inform adaptations of similar programs in different contexts. Further research is needed to develop a deeper understanding of how program components influence social health and longer-term behavior change.

## Introduction

### Background

There are 8.8 million US veterans aged >65 years [1]. Approximately one-third of older veterans have ≥3 chronic medical conditions [2]. This population represents nearly 90% of older veterans receiving care from the Veterans Administration (VA) [3]. The consequences of multimorbidity can be profound, influencing many aspects of an individual’s life. Multimorbidity is associated with impaired physical function [4], loneliness [5], high health care use, adverse health events (eg, falls and hospitalizations), and mortality [2,6,7]. These factors are also associated with poor health outcomes. For example, impaired physical function contributes to reduced walking ability, increased fall risk, and reduced participation in physical activity [8-11], and older veterans with multimorbidity are at a higher risk of social isolation and loneliness, which are associated with depression and mortality [12,13].

Although the VA has multiple established telehealth programs to assist veterans, no existing telehealth physical rehabilitation programs incorporating technology supports are tailored to older veterans with multimorbidity and complex health needs. For example, the MOVE! program is a weight management program that focuses mostly on nutritional education and coaching. The MOVE! program encourages routine physical activity as a strategy to support weight management; however, this program may not meet the needs of veterans with limitations in physical function, and not all locations offer exercise classes. Similarly, the VA Gerofit program is an exercise program designed for veterans aged ≥65 years, but the synchronous video telehealth version of the program requires veterans to demonstrate independence with exercises in a group setting, with or without caregiver assistance, and excludes those who require supplemental oxygen. As such, many veterans with multimorbidity and actual or perceived barriers to exercise are unable to participate in Gerofit.

Traditional physical rehabilitation programs can help prepare veterans to transition to community programs such as MOVE! and Gerofit; however, traditional episodes of care involve few sessions and often end before veterans are able to make such a transition. We sought to fill this gap by developing and evaluating a multicomponent telehealth program designed to address the complex needs of veterans with multimorbidity, teaching skills to support behavior change, and improving physical function so that the transition to community programs could be possible.

### Purpose

The purpose of this qualitative program evaluation was to describe the experiences of a multicomponent telehealth program and to identify opportunities for quality and process improvement. We conducted qualitative interviews with telehealth program participants to (1) explore their experience of individual program components; (2) document perceived outcomes and the impact on their daily life, relationships, and quality of life; and (3) collect feedback on this novel program to inform adaptations.

## Methods

### Program Evaluation Design

We conducted a multimethod program evaluation of our telehealth program. Quantitative results will be published separately. The focus of this study is to detail qualitative findings in relation to participant satisfaction, feedback, experience with key program components, and self-reported outcomes. This study follows the Consolidated Criteria for Reporting Qualitative Research guidelines [14] and the Standards for Quality Improvement Reporting Excellence reporting guidelines [15].

### Program Setting and Evaluation Group

The telehealth program was conducted as a joint project between the VA Eastern Colorado Healthcare System’s Geriatric Research Education and Clinical Center and the University of Colorado. All physical therapists were doctoral trained, 2 were board-certified geriatric clinical specialists, and 1 was completing a Geriatric Clinical Specialist residency.

The members of the program evaluation group included 2 clinical researchers (LMA and JES-L), 2 qualitative researchers (MLM and KAN), 1 physical therapist (MRR), and 1 research assistant. KAN also has >25 years of experience conducting program evaluations, including state-wide and national evaluations. Neither qualitative researcher had a therapeutic relationship with key informants to minimize the potential for bias.

### Theoretical Frameworks and Conceptual Model

**Figure 1:**
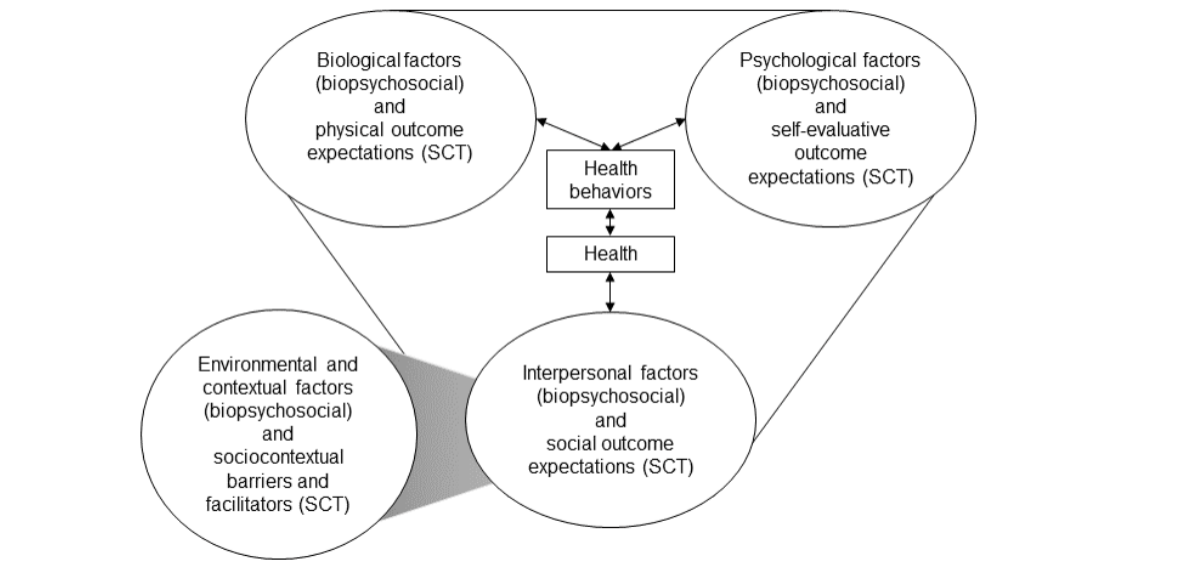
Conceptual model for the multicomponent telehealth program. SCT: social cognitive theory.

We combined 1 framework and 1 theory to develop a conceptual model for the multicomponent telehealth program (Figure 1). A conceptual model identifies key theoretical constructs that inform research questions and program design; it is common practice to combine multiple frameworks and theories [16]. Our conceptual model (Figure 1) was based on the biopsychosocial framework and social cognitive theory (SCT). The biopsychosocial framework [17] conceptualizes general health as the result of influences from biological, psychological, interpersonal, environmental, and contextual factors. This framework also considers health behaviors to fall under the umbrella of overall health; thus, behaviors are influenced by each domain. The biopsychosocial framework does not explicate the mechanisms of behavior change; therefore, we integrated SCT [18] into the conceptual model. SCT identifies the core concepts and mechanisms through which they contribute to behavior change. The concepts represented in our conceptual model included outcome expectations and sociocultural barriers and facilitators.

### Multicomponent Telehealth Program

We used our conceptual model to develop a 4-component telehealth program that addresses the complex and multifactorial needs of older veterans. Table 1 connects the theoretical constructs of our conceptual model to example problems, unmet needs, and their respective intervention components. Table 1 displays the sample interview questions related to each concept.

The 12-week multicomponent program was delivered by licensed physical therapists and a physical therapy assistant. The four core components of the program were (1) high-intensity rehabilitation [19] delivered during hour-long individual and group physical therapy sessions, (2) biobehavioral interventions [20-22] (termed *coaching*) delivered using motivational interviewing techniques, (3) social support during group physical therapy sessions, and (4) technology to augment intervention delivery (synchronous videoconferencing) and enhance participation (text messaging, activity monitor, and data sharing). Multimedia Appendix 1 [19,20,23-25] provides additional details about the program.

**Table 1:** Alignment of theoretical constructs from our conceptual model with program components and evaluation questions.

Theoretical construct	Example problems and unmet needs	Intervention	Interview questions
Biological (biopsychosocial) and physical outcome expectations (SCT^a^)	Multimorbidity Muscle weakness Reduced walking ability Falls	Component 1: progressive, high-intensity rehabilitation	What was the most meaningful change you saw in yourself?
Psychological (biopsychosocial) and self-evaluative outcome Expectations, self-efficacy, and goals (SCT)	Mental health illness Lacking skills to support behavior change	Component 2: biobehavioral intervention (“coaching”)	What did you think of the coaching sessions overall? How did the coaching sessions affect your exercise or activities during the program?
Interpersonal (biopsychosocial) and social outcome expectations (SCT)	Loneliness Social isolation	Component 3: social support via group physical therapy sessions	What was the experience of exercising in a group but virtually like for you? How did you feel in the group session?
Environment and context (biopsychosocial) and barriers and facilitators (SCT)	Reduced access to in-person care	Component 4: technology supports	What was it like to participate in an exercise program remotely? Tell me about some of the challenges you experienced during the program. How did you use your Fitbit? Tell me about your experience with the Annie text messages.
Health behaviors: physical activity (biopsychosocial and SCT)	Reduced participation in routine physical activity	Components 1-4	Think back on what a typical day looked like for you prior to this program and think about what your typical day looks like now. What is different? What is the same?

^a^SCT: social cognitive theory.

### Equipment

Participants were provided with exercise equipment, vital sign monitoring equipment, and a Fitbit Versa 2. All participants received at least 5 THERABAND resistance bands (yellow, red, green, blue, and black) and adjustable ankle weights. Participants received other exercise equipment such as an aerobic step, when needed. All participants who did not own vital sign monitoring equipment were provided with a pulse oximeter and an automatic blood pressure monitor. Participants who did not have a video-capable device received one through the VA’s digital divide program. A physical therapist taught participants how to use the exercise and vital sign monitoring equipment during a program orientation session, and an assistant taught participants how to set up the Fitbit.

### Key Informant Selection

Individuals were purposively sampled from a cohort that participated in the telehealth program. The eligibility criteria for the telehealth program were as follows: veteran and veteran spouse, age ≥50 years, ≥3 medical comorbidities, and self-reported reduced physical function (eg, difficulty walking and difficulty getting in and out of a chair). Any chronic medical condition listed in the participant’s chart was included in the total number of comorbidities; duplicative and similar entries were counted only once; for example, low back pain, degenerative disc disease, and lumbar stenosis were counted as 1 medical condition. Veterans were excluded if they had severe dementia (Montreal Cognitive Assessment [MoCA]-Telephone score <11), life expectancy <12 months, or a medical condition that would preclude safe participation in high-intensity rehabilitation (eg, unstable angina). Veterans were eligible for semistructured program evaluation interviews if they experienced all 4 components and participated in at least 8 weeks (out of 12 total weeks) of the telehealth program. A member of the team (MRR) verbally consented eligible participants.

### Data Collection

In total, 60-minute semistructured interviews were conducted between March and September 2021 using VA Video Connect, a secure videoconferencing platform. This platform was also used for all telehealth program sessions. The interviews involved 1 veteran and 2 program personnel (KAN and MRR or a research assistant). Interviews were conducted primarily by KAN, whereas MRR was present to take notes and ask detailed questions pertaining to the technology components of the program. Although MRR had a therapeutic relationship with some key informants, we encouraged candid feedback by explaining that the goal of the interview was to improve the telehealth program by learning about participants’ experiences.

An interview guide was used to focus the interview on veterans’ experiences of the program as they related to our objectives (Multimedia Appendix 2). The guide was developed using an iterative, team-based approach and was based on both program evaluation expertise and the conceptual model used in the program’s development [18,26]. Interviewers took detailed notes, and the interviews were audio recorded, professionally transcribed, and checked by a member of the study team for accuracy. The 2 interviewers completed a structured debrief [27] after each interview to identify new or emerging insights, discuss consistency with previous data, and reflect on the interview process to determine the necessary refinements to the interview guide.

Patient information, including rural status, miles saved, age, biological sex, BMI, functional comorbidity index [28], race, ethnicity, highest education, military branch of service, cognitive status, and mobile device proficiency, was collected. The Federal Office of Rural Health Policy Eligible Zip Codes file [29] was used to determine rural status. Miles saved was defined as the driving distance from the veteran’s physical address to the nearest VA facility. Cognitive function was assessed using the Telephone MoCA. This test is a modified version that removes items requiring vision; therefore, it may be conducted via phone. Scores ranged from 0 to 22, with scores ≥18 considered nonimpaired [30]. Baseline technology skills were assessed using the Mobile Device Proficiency Questionnaire-16; this 16-item survey measures a person’s capability to perform different tasks using a mobile device using a 1 to 5 Likert scale (1=never tried, 2=not at all, 3=not very easily, 4=somewhat easily, and 5=very easily). Summative scores ranged from 8 to 40, and scores ≥32 indicated a mean score of somewhat easy for each task domain [31].

### Data Analysis

A directed content analysis approach [32] was used to analyze the qualitative data. MRR and KAN developed the initial codebook based on salient theoretically informed constructs and codes that emerged from structured debriefs (oral and written). Deductive codes were derived from the theoretical frameworks. For example, *self-efficacy* was a salient construct from SCT [18], and social support was informed by the interpersonal factors construct of the biopsychosocial framework [17]. The codebook was loaded into Dedoose (SocioCultural Research Consultants, LLC) software to manage and code all interview data. MRR and MLM independently coded the first few transcripts and then met to discuss the findings and resolve discrepancies. Interrater reliability was established when the final independently coded transcript did not show any discrepancies. MRR, MLM, and KAN met as needed during the analysis to discuss conceptual domains, reorganize the codebook, and identify themes. Once the final codebook was established, MRR and MLM coded the remaining transcripts.

### Ethical Considerations and Participation

Our institutional review board determined that this study did not meet the definition of human subjects research. The participants in the pilot program received a Fitbit Versa 2 for compensation.

## Results

### Sample Characteristics

A total of 21 individuals enrolled in the program; 15 individuals completed at least 8 weeks, meeting the inclusion criteria for interviews, and all 15 individuals agreed to participate. In total, 6 individuals withdrew from the program before 8 weeks, and thus, they were not eligible or available for interviews. The reasons for withdrawal included medical decline (n=3), work conflicts (n=2), and loss to follow-up (n=1). Interviews lasted for a mean of 60.4 (SD 8.2) minutes. Most interview participants (11/12, 92%) completed the 12-week program; 1 individual completed 8 of the 12 weeks but did not finish because his pain worsened, and he decided to seek alternate treatment options. Mean age was 65 (SD 9.4) years; one-quarter of the participants were from rural areas, and three-quarters of the participants were male. One-third of the interview participants had Telephone MoCA scores suggestive of mild cognitive impairment. Additional demographic data are presented in Table 2.

**Table 2:** Key informant characteristics (n=15).

Characteristic		Values
Rural (yes), n (%)		4 (27)
**Miles saved, median (IQR)**		
	Per 1 visit	30 (10-48)
	Total	966 (75-1057)
Age (years), mean (SD)		64.5 (9.4)
Biological sex (male), n (%)		11 (73)
BMI (kg/m^2^), mean (SD)		28.6 (5.0)
Functional comorbidity index, mean (SD)		4.9 (2.5)
**Race, n (%)**		
	Black or African American	2 (13)
	White	9 (60)
	More than one	4 (27)
**Ethnicity, n (%)**		
	Hispanic or Latinx	2 (13)
	Not Hispanic or Latinx	13 (87)
**Education, n (%)**		
	High school or GED^a^	1 (7)
	Some college or associate degree	7 (47)
	Bachelor’s degree	3 (20)
	Postbaccalaureate	4 (27)
**Military branch, n (%)**		
	Air Force	7 (47)
	Army	4 (27)
	Navy	3 (20)
	Nonveteran, veteran’s spouse	1 (7)
**Telephone-MoCA^b^**		
	Score (0-22), mean (SD)	17.9 (2.5)
	Impaired cognition (<18/22), n (%)	5 (33)
MDPQ^c^-16 (8-40), mean (SD)		34.5 (7.4)

^a^GED: Tests of General Education Development.

^b^MoCA: Montreal Cognitive Assessment.

^c^MDPQ: Mobile Device Proficiency Questionnaire.

### Domains, Codes, and Themes

#### Overview

A total of 6 main conceptual domains were identified, each aligned with ≥1 of the key program components or goals of the program evaluation: technology, social network, therapeutic relationship, personal attributes, access, and feasibility. Table 3 presents the final coding structure organized by the theoretical constructs and domains and the operational definitions of each code. Table 4 provides illustrative quotations.

**Table 3:** Theoretical constructs, domains, codes, and definitions.

Theoretical constructs, domain, and code				Definition
**Environment and context (biopsychosocial) and barriers and facilitators (SCT^a^)**				
	**Technology**			
		Competency	The ease with which an individual was able to use a specific technology	
		Satisfaction	The degree to which an individual enjoyed using and found value in a specific technology	
		Accountability	A sense of responsibility for one’s actions that arose from using a specific technology	
**Interpersonal (biopsychosocial) and social outcome expectations (SCT)**				
	**Social network**			
		Internal social support	An individual’s relationship with people inside the program, excluding their physical therapist. Peer comradery: the sense of belonging based on shared experiences Friendly competition: competition that arises when working toward a common goal and allows all those involved to experience enjoyment or pleasure	
		External social support	An individual’s relationships with people outside of the program, for example, friends and family	
**Interpersonal (biopsychosocial) and social outcome expectations (SCT)**				
	**Therapeutic relationship**			
		Trust	The quality of belief in a clinician’s reliability, truth, or strength as it pertains to the care of the patient. The extent to which the patient believes the clinician considers their best interests	
		Clinician qualities	Personal or professional traits of the clinicians and other staff involved in the program	
		Communication	The quality and content of verbal and nonverbal communication either directly experienced between the clinician and the patient or perceived to occur among the clinicians and staff	
		Personalization	The extent to which the program was tailored or adapted to meet the specific needs and goals of the individual	
**Psychological (biopsychosocial) and self-evaluative outcome expectations, self-efficacy, and goals (SCT)**				
	**Personal attributes**			
		Motivation	Internal or external reasons, needs, or desires to either maintain or increase physical activity behaviors (or other health behaviors such as sleep habits)	
		Attitudes and beliefs	Included current and past attitudes and beliefs about pain, physical activity, and exercise	
		Expectations for physical therapy	The beliefs that certain physical therapy interventions would be used during sessions; beliefs may be based on prior experiences of in-person physical therapy episodes of care	
**Environment and context (biopsychosocial) and barriers and facilitators (SCT)**				
	**Access**			
		Equipment availability	Perception of access to the equipment necessary for program participation and the experiences using the equipment during the program	
		Efficiency	The degree to which the burdens of attending physical therapy were minimized	
		Convenience	The perceived ease of participating in physical therapy or being able to participate in physical therapy without having to make a significant effort to attend sessions	
		Solution to barriers precluding in-person care	Reasons identified that would have precluded the ability to attend in-person physical therapy	
**Program-specific health behaviors: physical** **activity (** **biopsychosocial** **and SCT** **)**				
	**Feasibility**			
		Program logistics	The perception of the program logistics considered as a whole (eg, session frequency and duration) and for each separate program component (eg, individual sessions, group sessions, and coaching)	
		Preparedness	An individual’s readiness to begin group physical therapy sessions	
		Perceived benefits of program participation	The benefit or benefits, or lack thereof, experienced by veterans. These outcomes may have occurred in any area of their health—physical, mental, social, or behavioral	

^a^SCT: social cognitive theory.

**Table 4:** Domains, codes, and illustrative quotes.

Domain and code			Illustrative quote (*theme*^a^*)*
**Technology**			
	Competency	“The Fitbit was simple and easy to use.” [Veteran #226] (*Competency supported by ease of use*) “I struggled with [VVC^b^]. For about three times, three weeks, I had a hard time connecting.” [Veteran #208] (*Lack of previous experience led to longer learning time to develop competency*)	
	Satisfaction	“But the Fitbit I like because I like to track the number of steps I take.” [Veteran #208] (*Activity tracking enhanced by technology—a perceived benefit*) “I don’t really care how many steps I take...and when it said I didn’t meet 10,000, I’d just go, ‘So,’ and go about my business. I didn’t really understand the reason for the Fitbit if you want the truth.” [Veteran #227] (*No perceived value leading to dissatisfaction*)	
	Accountability	“I liked that [Annie] was consistent, that I knew at a certain hour, I was like, ‘Oh my gosh, I need to get hot because Annie’s gonna be asking me how many steps I did for the day.’” [Veteran #105] (*Enhanced accountability achieved through technology*)	
**Social network**			
	Internal social support	“It became almost like a family. You know, I tried to treat it that way because we’re Vets, you know? We were – we’re all trying to do the best that we can.” [Veteran #207] (*Shared experience of being veterans contributed to peer comradery*) “It’s not really much of a group when there are just two people. And [there] really was no ability to really interact with the person.” [Veteran #217] (*Experience of a small group and structure resulted in a lack of peer comradery*)	
	External social support	“[My daughter] needed somebody to go with her, and I wanted to go back [to the gym], especially since I’d gone through the PT.” [Veteran #227] (*Plans to continue exercise included key exercise buddy*)	
**Therapeutic relationship**			
	Trust	“I made it all the way to the end [of the program] because it was working, and I trusted [my physical therapist].” [Veteran #109] (*Trust in therapist facilitated program engagement*) “I think that had it gone longer, where you could probably get some real benefit is that relationship with that physical therapist in that group setting.” [Veteran #225] (*Not enough time to establish trust*)	
	Clinician qualities	“[PT] was motivating, she was funny, encouraging, and I think it was an important aspect of this to say, ‘Somebody cares about you when you’re not in session.’” [Veteran #211] (*Clinician qualities that enhanced the therapeutic relationship*)	
	Communication	“If [the clinicians] felt there was any issue with personal safety, it was like, you know, ‘Make this correction, protect yourself.’” [Veteran #104] (*Demonstrated attention to safety enhanced the therapeutic relationship*) “When you have different physical therapists, there’s inconsistency.” [Veteran #223] (*Perceived lack of provider-to-provider communication negatively influenced the therapeutic relationship*)	
	Personalization	“They modified really quick, and until I could do something that didn’t hurt too bad.” [Veteran #227] (*Exercise modification to reduce pain enhanced the therapeutic relationship*) “[During the coaching] we talked about, What are my goals? What is it that I want to achieve? We talked about the whys.” [Veteran #109] (*Coaching support provided opportunities for personalization*)	
**Personal attributes**			
	Motivation	“I’m limited on what I can do now, and I don’t want to be any more limited than what I am.” [Veteran #221] (*Internal motivation to prevent physical deconditioning*) “I wanted to dance, and I wanted to bowl, and I wanted to do those things.” [Veteran #211] (*Internal motivation to reengage in enjoyable activities*) “The best part [of the program] was getting me motivated to get some exercise and to get moving again.” [Veteran #217] (*Participation in the program served as an external motivator*)	
	Attitudes and beliefs	“Exercise is, you might say, a priority now.” [Veteran #228] (*New attitude about exercise*) “Exercising before this program was vital to me.” [Veteran #104] (*Consistent with previously held attitudes and beliefs about exercise*) “I didn’t know how to exercise without hurting myself, so I learned that.” [Veteran #211] (*Experience in program facilitated change in previously held fear-avoidance beliefs about exercise and pain*)	
	Expectations for physical therapy	“There has always been a physical inspection and manipulation stage of therapy that didn’t and couldn’t happen here [through telehealth].” [Veteran #226] (*Experience inconsistent with expectations and previous experience*) “‘This is what you’re doing, this is what you need to do, get at it.’ That’s PT. You’re being told how to get yourself better.” [Veteran #221] (*Expected a direct communication style from the physical therapist based on previous experience*)	
**Access**			
	Equipment availability	“[The staff has] everything covered. I mean, with the blood pressure thing and the Fitbit and the pulse ox. And the other stuff, the equipment, I thought it was great, I really did.” [Veteran #227] (*All necessary equipment was provided*)	
	Efficiency	“A one-hour [in-person] appointment turns into a two-and-a-half-hour waste of my day. This [telehealth] is one hour, and I’m out of here.” [Veteran #226] (*Time efficiency that facilitated access*)	
	Convenience	“[The telehealth program] was, by far, the easiest to do. It fit in with my schedule nicely.” [Veteran #226] (*Scheduling convenience that facilitated access*) “I don’t have to travel to have physical treatment. I can do it in my own home.” [Veteran #210] (*Convenience of location that facilitated access*) “Where it came apart was when they wanted me to do group sessions, and there was only that dedicated time. That made it difficult on me because those times weren’t necessarily most conducive in my world.” [Veteran #223] (*Inconvenience of scheduling that impeded access*)	
	Solution to barriers precluding in-person care	“I have cataracts in my eyes. I can drive around the neighborhood, but I can’t drive across town to an appointment for therapy.” [Veteran #210] (*Physical health barrier to in-person care*) “In the interest of flexibility and safety with the current environments that we’re existing in [referring to the COVID-19 pandemic], and I also have a panic anxiety disorder, so I don’t do well in big groups, at times. So not having to go into the rehab facility and being around a whole lot of people was very conducive, as well.” [Veteran #223] (*Mental health barrier to in-person care*)	
**Feasibility**			
	Program logistics	“Some weeks [the session frequency] didn’t matter at all. It was fine, and I could do it. There were other weeks where it was just—I couldn’t make all of those.” [Veteran #226] (*Competing priorities prevented attendance at sessions*)	
	Preparedness	“We would learn the exercises in the individual [sessions]. It would be reiterated the second session on the Wednesday, and then on Friday when it’s introduced in group, she would also go over it.” [Veteran #109] (*Feeling prepared for group sessions through repeated practice during individual sessions*)	
	Perceived benefits of program participation	“I’m stronger. I’m a lot more limber.” [Veteran #227] (*Physical health benefit*) “Instead of driving to the store, there’s a store, like, five blocks up, so I’ll walk to the store.” [Veteran #109] (*Beneficial change in physical activity health behavior*) “This program has changed the quality of my life because she gave me some stretches and some moves that has now made my back stop hurting. My back has been hurting since 2011.” [Veteran #109] (*Reduced pain led to improved quality of life*)	

^a^Italics indicate the theme associated with the preceding illustrative excerpt.

^b^VVC: VA Video Connect.

#### Domain 1: Technology

##### Competency

Interview participants noted varying levels of technology competency, which were influenced by previous experience, self-efficacy in learning and using new technology, and the degree to which they had access to social support. All veterans were required to join synchronous videoconference sessions using the VA Video Connect platform for all program sessions. Participants reported an improved ability to log into these appointments over time. For example, one participant shared the following:

By the time you get on with your [physical therapist] person, it’s real fluid. I can [log-on to VA Video Connect] in, like, two minutes now.

Veteran #211

Often, veterans found one technology easier to use than others, with the Fitbit and Annie being among those used most often to monitor progress. Participants reported a variety of methods for setting up and maintaining technology tools. One veteran shared, for example:

The Fitbit was easy. I said, “Here, honey [referring to his wife], program this for me”

Veteran #211

Help-seeking resources included asking family members for help, as seen in the quote from veteran #211, calling dedicated helplines (eg, the VA National Telehealth Helpdesk), and searching the internet for videos or articles. Our team also provided orientation sessions to help participants set up and become familiar with technology tools and provided ongoing support.

##### Satisfaction

The level of satisfaction varied even for the same individual, depending on the specific technology being discussed. For example, veteran #221 was ambivalent about Annie—*“*Sometimes [Annie] was annoying and sometimes it was helpful”—but was satisfied with the Fitbit—“I love the sleep part of [the Fitbit] ‘cause it tells me how my sleep is going. I see my sleep doctor once a year, and now I can actually share this information with them to let them know how I am sleeping.” Participants tended to be satisfied with a specific technology when they associated its use with a benefit. This value-driven satisfaction was evident when veterans discussed how the technology fostered a sense of encouragement to be active or held them accountable to the program and their goals:

Like it was Team [me]. Like, [Annie] was my little other coach. So, like, she was my third coach

Veteran #105

Some veterans expressed dissatisfaction with the various technologies; this feeling often emerged when there was no value seen in the technology. For example, a veteran did not find value in the text messages from Annie:

For me, Annie was nothing but annoying. I did not need encouragement from Annie.

Veteran #226

At other times, dissatisfaction was expressed when using the technology was inconvenient or required extra steps:

I can’t tell you how thrilled I was when [the text messages] stopped. It was just an irritation that I had to take time, go to another screen, and look to find out the information I needed to know, connected with Annie.

Veteran #217

##### Accountability

Statements of accountability emerged primarily when veterans were discussing the Fitbit and Annie. Some veterans noted how the Fitbit helped to provide accountability because it became a source of feedback on a person’s daily activities by providing them with data. Having knowledge of these data allowed veterans to set goals and monitor their progress, fostering a sense of personal accountability. Annie most often provided accountability through the consistency of the text messages asking for daily step counts:

I knew that at 8:00 and 11:00 every night, Annie would be wanting my steps.

Veteran #220

When veterans set daily step count goals, the Fitbit tracking and Annie reporting functions seemed to work synergistically to keep veterans accountable.

#### Domain 2: Social Network

##### Internal Social Support: Peer Comradery and Friendly Competition

The program staff sought to intentionally foster peer comradery during group physical therapy sessions. Most veterans noted that peer comradery was present and often facilitated through the shared experience of being veterans; this comradery enhanced their experience of the program overall. Peer comradery, when present, can support the emergence of friendly competition among participants, often motivating them to increase their effort:

I tried to do more than these guys—the young guys, you know, the 55- and 60-year-olds.

Veteran #208

Not all participants who described comradery also described this experience of friendly competition, a finding that suggests that this perception was also tied to a veteran’s personality, prior experiences, or group dynamics.

In contrast, some veterans described the absence of comradery. This lack of comradery was influenced by one or more of the following factors: (1) the veteran experienced a small group of only 1 to 2 people owing to slow program enrollment, (2) the group comprised veterans at very different levels of physical function, or (3) the veteran perceived a lack of personality fit. The following quote illustrates the absence of comradery stemming from having varying levels of physical function and personalities within different groups:

Part of the problem for me, too, is that I was either grouped with people...with a lot more limitations and adaptions that needed to be done and older and more frail situations, it seemed. The second one was strictly your kind of proverbial GI Joe, and he was not a very pleasant GI Joe. And so, it just was not a good mix because I'm somewhere else in the spectrum

Veteran #223

These differences also contributed to feelings of frustration if a veteran was unable to participate at a desired intensity, for example, when patients with higher physical function and capacity needed to slow down or have longer rest breaks to accommodate veterans with lower physical function and capacity.

##### External Social Support

Some participants described having close familial relationships, which they indicated were instrumental throughout the program, for example, by helping participants manage the technical aspects of participation. Relationships outside the program often served as additional sources of motivation for program participation, especially when spouse- or family-centered activities were important to the individual:

[My wife and I] play music in the kitchen, and when we’re cooking, we dance together. I couldn’t do that [before the program]...I mean, just to be able to enjoy our company the way we had before my knee started going so bad, was -- hey, life’s a great pleasure.

Veteran #211

These external relationships also played a role in the plans to continue exercise.

#### Domain 3: Therapeutic Relationship

##### Trust

Most participants identified trust as the keystone of the therapeutic relationship, and trust played a critical role in their overall experience of the program. Veterans noted that their therapists established trust through various mechanisms. One method was by proactively addressing potential safety hazards in the home environment and by being responsive to patients’ concerns:

There wasn’t [a time I felt uncomfortable doing an exercise] because the therapists made sure that we had a chair, a grab rail, something there in case we lost our balance.

Veteran #221

Veterans perceived trust as a facilitator for program engagement. Sometimes, trust was not established with clinicians, and perceptions of not having enough time to work with a clinician or clinicians other than their primary individual physical therapist surfaced as a barrier to the emergence of trust. Clinicians’ qualities, communication, and personalization have also contributed to the development of trust.

Veterans viewed trust as bidirectional, in that the clinician also needed to demonstrate trust in the patient and their judgment. One participant identified how she perceived that her physical therapist had trust that she would follow through with the plan of care when she needed to transition to an asynchronous telehealth approach because she was no longer able to attend the scheduled group sessions:

I knew that I had established trust with [my physical therapist] and that we also had formulated a really good connection, and that she was wanting to see my success as much as I wanted to stay in the program.

Veteran #105

In contrast, 1 veteran provided an example of how he felt the clinician did not trust his interpretation of pain during exercise. He stated the following with exasperation:

I said, “Oh, that one—that one caused some pain. That was a good work out.” And [the clinician leading the group session stopped] the whole dang thing ‘cause I used the trigger word pain. Just ‘cause you feel it, doesn’t mean you’re hurt. Just ‘cause there’s pain, doesn’t mean you’re injured!

Veteran #226

This excerpt also identifies the importance of communication between the patient and the clinician.

##### Clinician Qualities

Clinicians’ qualities contributed to the therapeutic relationship, and key informants often described their therapists as motivating, caring, informative, encouraging, kind, professional, and attentive:

That’s the advantage to having someone paying attention to what you’re doing and trying to convince you to do better, as well as having a whole program where you’ve got some caring people that encourage you to do better.

Veteran #220

Veterans often expressed that their coaches and therapists were a source of accountability:

[The coach] put into perspective my part ‘cause it gave me accountability.

Veteran #109

These positive clinician qualities helped establish a strong therapeutic relationship and made patients feel valued and respected:

I have never had this kind of care in my life...I have never had this good of care period.

Veteran #227

##### Communication

Most participants felt that there was beneficial and constructive communication, especially with their individual physical therapist or coach. Collaborative communication was often key in developing the therapeutic relationship:

A lot of providers can be very cold. It’s just like, you know, people are talking at you instead of talking to you. And at no time during the course of this program did I feel that anybody was talking at me. They were really concerned for me and for me, uh, improving, being better, getting stronger.

Veteran #104

Veterans appreciated hearing about their progress during reassessments and gaining new knowledge about exercise and movement. However, there were also instances when there was a lack of communication, and although this may not have been perceived as a negative experience, the participants viewed such instances as a missed opportunity. For example, 1 veteran noted the following:

We’re meeting Monday, Wednesday, Friday. On Tuesday and Thursday and then the weekends, this is what I want you to do. That instruction was lacking.

Veteran #226

Veterans also noted that most communication deficiencies occurred during group sessions, especially if the physical therapist leading the group was not the same clinician who led their individual physical therapy sessions. In some instances, this created an environment that fostered a negative experience for veterans:

This [physical therapist] didn’t really know me from anybody, had no foundation with me. So, if they don’t know me, how can they suggest what is a good exercise for me?

Veteran #223

We also heard similar experiences when veterans saw multiple therapists throughout the program.

##### Personalization

Individual physical therapy sessions, which occurred before the transition into group sessions, facilitated the therapist’s ability to tailor the exercise program to each participant’s needs and goals. Sometimes, this tailoring involved connecting certain exercises to a patient’s motivation for participation; for example, the squat exercise is used to build leg strength so that for one patient, it will be easier to get in and out of a chair, and for another patient, it will be easier to pick up their grandchild. At other times, personalization required the therapist to identify an alternate exercise to address certain muscle groups without exacerbating pain. For example, if the goal is to strengthen the quadriceps muscles, but a standing squat is painful, this exercise could be modified to a seated knee extension; this facet of personalization was important for facilitating changes in attitudes and beliefs (detailed below in *Domain 4: Personal Attributes* section) and developing trust:

In the individual sessions when I expressed my concerns about any type of movement, and then [the physical therapist] showed me a modification and they worked, what it did was it allowed me to be able to trust her.

Veteran #109

Coaching sessions were naturally personalized because the primary focus was to elicit the patient’s motivations for change and establish goals.

#### Domain 4: Personal Attributes

##### Motivation

Veterans identified various intrinsic motivations that included a desire to improve physical health or prevent further deconditioning and loss of mobility. Sometimes, a key motivation was a desire to reengage in valued activities such as golf, dancing, or bowling. For some veterans, internal motivation was strongly related to their personality or identity:

There was no problem with being motivated or anything else ‘cause, truthfully, nobody’s gonna out work me in what I decide.

Veteran #227

Some veterans discussed their need for extrinsic motivation, such as being held accountable by their health care clinician to start and complete the program or using technology such as the Fitbit, which provided different acknowledgments for achieving activity goals. Most veterans described both types of motivations.

##### Attitudes and Beliefs

The veterans described the current and past attitudes and beliefs related to exercise and pain that influenced their experience of the program. Most patients identified that exercise was or had become an important part of their life. For some veterans, this was a new attitude, whereas for others, it was a persistent belief, and the program served as a source of support for reestablishing regular exercise habits. Some veterans who were hesitant to exercise before the program owing to pain realized that there were alternative ways to exercise without experiencing pain; this was a key mechanism through which therapists enhanced the therapeutic relationship while also helping patients break out of fear-avoidance behaviors. Some patients also described learning the difference between expected muscle soreness (eg, *good* pain) versus joint pain (eg, *bad* pain), helping to learn or reinforce body awareness while minimizing fear of injury:

Once I realized that it’s just being sore and not—I’m not hurting myself, it, you know, all is well.

Veteran #109

In contrast, some veterans had a “no pain, no gain” attitude toward exercising before starting the program. They learned how to reframe this mentality and listen to their body’s cues:

The program helped me in so many ways learning to listen to my body, learning to accept that my body has limitations. My brain doesn’t want to accept it, but I’ve learned to acknowledge that the body says, “No, we can’t do this,” and it’s all right.

Veteran #221

##### Expectations for Physical Therapy

Finally, expectations played a significant role in how participants experienced the overall program and perceived the quality of the care they received. Such expectations were often related to prior physical therapy experiences. For example, interview participants noted aspects of care that were absent in the telehealth program compared with their prior in-person physical therapy experiences. Specifically, some veterans noted that there was a lack of “hands-on” interventions such as manual therapy (eg, soft tissue massage or joint mobilization) and physical examination (eg, palpation). Care expectations may have contributed to the difficulty that some participants described engaging in the coaching sessions:

We sat there and talked, and maybe because I wanted to do things—I didn’t want to just talk for a half hour—and so that’s why maybe I didn’t get anything out of [the coaching].

Veteran #208

Some patients also expected a more traditional communication style during physical therapy (ie, directive) rather than the guiding and following communication styles used as part of the motivational interviewing techniques. Interestingly, some veterans noted a need for self-advocacy in the telehealth setting, which may not need to happen to the same extent during in-person physical therapy:

The Veteran has to own a little bit of responsibility for sure. If I’m being a big boy and doing this over the tele-rehab thing, I’ve got to be a big enough boy to look and see that there’s swelling that isn’t being addressed with ice and etc., and I need to bring that up.

Veteran #221

#### Domain 5: Access

##### Equipment Availability

The core equipment used during the program and provided to patients on an as-needed basis included monitoring equipment (automated blood pressure cuff and pulse oximeter) and exercise equipment (bands, ankle weights, and aerobic steps). The participants were trained on how to use their equipment and how to set up their exercise equipment safely in their home. Most veterans either had access to the necessary equipment at the beginning of the program or felt that we adequately provided what they needed. The patients shared their feedback on the equipment used. For example, 1 veteran stated that he enjoyed using the aerobic step. However, he did not feel safe using the risers to increase the height of the step because it seemed unstable to him. He also noted that it was difficult to maneuver at home because it was a large step.

##### Efficiency

Two key themes emerged in relation to efficiency: time efficiency and cost efficiency. Veterans identified time efficiency primarily as the reduced time required to travel to and from a facility to receive physical therapy. For some individuals who relied on public transportation or services such as Access-a-Ride for transportation, this was a significant time saving. Veterans also noted cost savings related to transportation, mostly stating that they did not have to pay for gas. The themes of time and cost efficiency were often intertwined for individuals who were working. These veterans described being able to participate in the synchronous videoconference exercise sessions either during their lunch hour or by adjusting their workday to accommodate the therapy sessions. They viewed the time saved as a cost saving because it meant taking less sick leave from work or preventing lost revenue for those who were self-employed.

##### Convenience

Participants often talked about convenience in relation to being able to receive physical therapy services on a day and time that worked for their schedule, thus facilitating access. Many veterans found that individual sessions were much easier to fit into their schedule than the group sessions. This sentiment was especially true among patients who were working full time because the group sessions occurred on set days and times and were thus more difficult to attend. For some participants, the inflexibility of the group sessions was a barrier to accessing this program component. Patients also appreciated the convenience of being able to perform physical therapy from their home—an environment that was logistically easier and, perhaps, more socially comfortable than other settings:

I like the format; I like the video. I hated going to the gym. I hated going to the fitness centers and things like that where everybody else is, you know, and going into the locker rooms. It was just a pain.

Veteran #220

##### A Solution to Barriers Precluding In-Person Care

This telehealth program offered a solution to barriers that might have otherwise precluded receiving physical therapy as in-person care. Some veterans described physical health conditions that made it difficult to leave their home or drive. Other veterans noted that mental health conditions, such as social anxiety, made it difficult to attend in-person physical therapy. Such anxiety may have impacted their willingness to continue participation over the 12 weeks had the program been in person. Finally, owing to the timing of this program during the COVID-19 pandemic (2021), many veterans were wary of spending extended time in public spaces when there were alternative options available. Concerns about COVID-19 were particularly salient given the medical vulnerability of the population. For these reasons, the telehealth program offered an attractive solution for receiving physical therapy services. For some rural veterans, telehealth was the only option to access physical therapy services through the VA because they lived too far away from a VA facility.

#### Domain 6: Feasibility

##### Program Logistics

Overall, veterans found the frequency and duration of the program to be appropriate, especially when there was value or benefit seen from attending the sessions:

There’s enough continuity that [the program] works well. You know, [if you’re] only doing it once a week I don’t know that we can see any substantial improvement in ourselves, but doing it, you know, three times a week and then having a counseling session to talk through goals and objectives...That, I think is great.

Veteran #104

Others were ambivalent about the program’s logistics, whereas some veterans identified the frequency of sessions and the duration of the program as burdensome. This perception often changed depending on competing priorities.

The extent to which individuals viewed the program’s separate components as manageable also related to the perceived value. There was a subgroup of veterans who did not find value in the coaching sessions; thus, they felt that these additional sessions were burdensome:

[The coaching sessions] were kind of lame and not helpful. Build goals. You know, for me it was frustration to have those sessions.

[Veteran #223]

##### Preparedness

Most veterans felt they were adequately prepared to make the transition from a higher level of supervision in the individual sessions to a lower level of supervision in the group sessions. The sense of preparedness was often a reflection of the quality and content of communication between the provider and the patient:

I think the people that worked with me knew what I should be ready for and started on the individual at the beginning and then working into the group. I know [my individual physical therapist], every once in a while, would say, “You’ll experience this one in group.”

Veteran #228

Interestingly, 1 veteran viewed the individual sessions as being more important for the clinicians to prepare for group sessions:

[The] individual session was more a preparation for them [clinicians] to deal with each of us individually than it was preparing me for a group session.

Veteran #211

A small group of veterans felt that the individual sessions did not prepare them for the exercises included in the group sessions:

None of the stretching that you learned in your individual sessions translate or moved over into the group session.

Veteran #225

Notably, this perception seemed to occur when the physical therapist deviated from the protocol, often attempting to better meet the patient’s individual needs.

##### Perceived Benefits of Program Participation

Overall, the perceived benefits facilitated program engagement. Most participants reported improvements in physical health, and some veterans also reported improvements in mental health such as improved mood and enhanced self-efficacy, for example:

I’m confident enough I can [walk on soft, uneven ground] because of the success in the program.

Veteran #211

**Figure 2:**
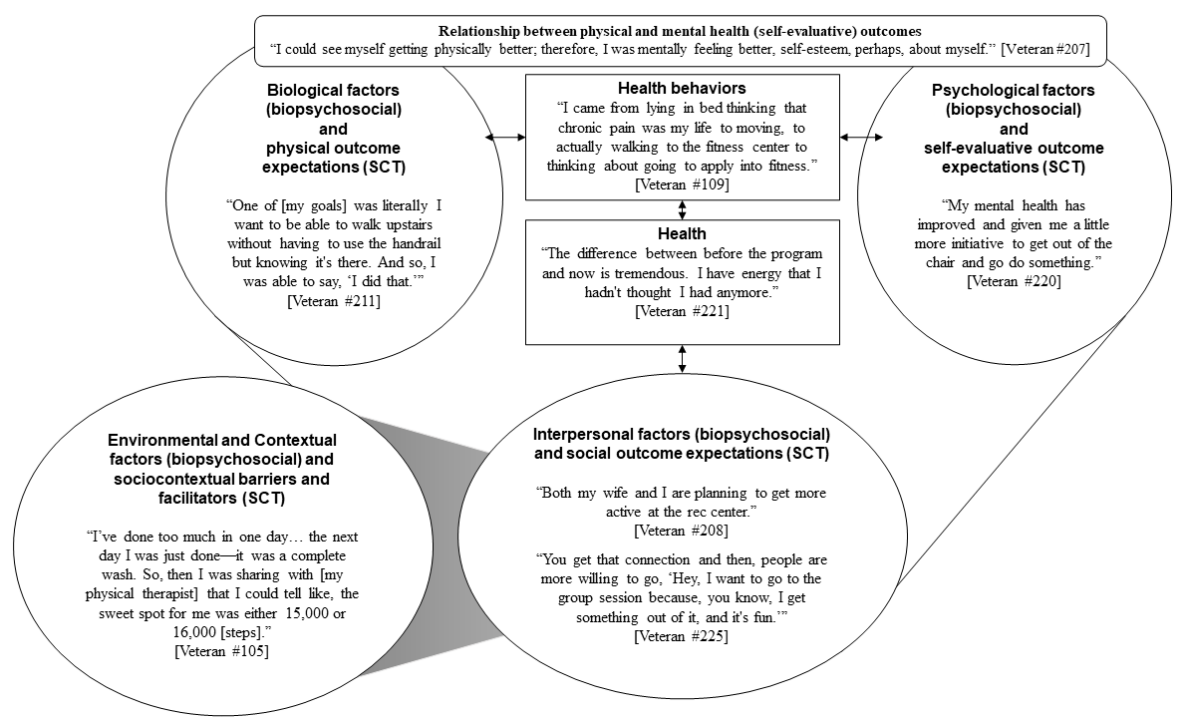
Alignment of key informants’ perceived benefits with the conceptual model for the multicomponent telehealth program. SCT: social cognitive theory.

Self-reported changes in physical and mental health were often related, and veterans noted that improvements in physical health enhanced both mood and mental health. One veteran also noted that before the program, visiting her children and grandchildren out of state was not possible for multiple reasons, including her physical health, chronic pain, and concerns about contracting COVID-19; however, following the program, she mentioned that she was considering traveling to see her children and grandchildren because she had less pain, was able to move around easier, and was able to get a COVID-19 vaccination. Figure 2 displays the perceived benefits in alignment with our conceptual model. Figure 2 also includes social outcome expectations (SCT), which were discussed in domain 2 (social network), and contextual factors (biopsychosocial and SCT), which were discussed in domain 1 (technology) to demonstrate the alignment of outcomes with our conceptual model.

Some veterans reported negative outcomes. Two veterans experienced injurious falls while they were enrolled in the program. Although both instances occurred outside the therapy sessions and were unrelated to program interventions, these injuries either delayed or prevented progress during the program:

Throughout the program, I was hurt, so that was somewhat of a hindrance.

Veteran #207

## Discussion

### Principal Findings

The purpose of this evaluation was to explore veterans’ experiences of a multicomponent telehealth program and identify opportunities for improvement. Key informants discussed positive experiences during the program, reinforcing specific program components and processes. Most key informants identified physical health improvements such as improved strength and energy, whereas some described mental and social health improvements including improved mood and the ability to return to valued activities. Key informants also described changes in or reinforcement of positive exercise attitudes and beliefs. Some veterans noted specific negative experiences, which informed program modifications.

We used our conceptual model to design the multicomponent telehealth program by connecting intervention components to theoretical constructs. Component 1, high-intensity rehabilitation, was related to biological factors (biopsychosocial) and physical outcome expectations (SCT). Veterans attributed physical health improvements—improved muscle strength, enhanced balance, and greater daily energy levels—to the high-intensity rehabilitation component of the telehealth program. Some participants expressed an intention to continue doing at least some of the exercises after program completion, demonstrating positive health behavior changes. There is robust evidence to support physical health improvements following various exercise interventions [33]. Furthermore, there is a dose-response relationship between exercise and outcomes, such that longer-duration programs (ie, 8-12 wk) are associated with more robust gains compared with shorter-duration programs [34].

Component 2, biobehavioral intervention (coaching), was designed to address psychological factors (biopsychosocial) and self-evaluative outcome expectations, self-efficacy, and goals (SCT). Key informants’ expectations for physical therapy seemed to influence their expectations regarding the content of coaching sessions. Those who expected coaching sessions to include traditional physical therapy interventions (ie, exercise) tended to perceive low value in these sessions. Perceived benefits from coaching included identifying motivations for change, enhancing self-efficacy, and goal setting. Prior research has shown that behavior change interventions, similar to our coaching intervention, often include these strategies [35,36]. Coaching sessions were sometimes viewed as burdensome, especially for program participants who had less understanding of the purpose or who saw little value in coaching.

Physical and mental health were mentioned concurrently by some veterans who noted that improvements in physical health improved mood and mental health. This relationship is supported by robust evidence showing that exercise improves mood in individuals with mental health conditions [37,38]. Some veterans noted changes in fear-avoidance behaviors that emerged through the therapeutic relationship via personalization and communication; for example, veterans who were hesitant to exercise owing to fear of causing injury or experiencing pain were able to overcome this negative outcome expectation. In addition, those who had a fear of falling experienced a reduction in this fear, as they gained more confidence in performing exercises and walking. These outcomes—fear-avoidance behaviors and fear of falling—were not part of our conceptual model and may be beneficial to measure quantitatively in future studies.

Component 3, group physical therapy sessions, was related to interpersonal factors (biopsychosocial) and social outcome expectations (SCT). Many veterans reported a feeling of comradery, which not only enhanced their participation but also served as a source of external motivation for some. These findings are consistent with studies of in-person groups that demonstrated enhanced motivation and adherence [39,40]. A longer-term goal of this program is to facilitate the transition into group-based community programs (eg, Gerofit, MOVE!, and Silver Sneakers). Some veterans shared intentions to transition into group-based exercise programs, whereas others planned to continue independently with a home program. These experiences support the potential of the program to fill the gap between traditional rehabilitation and community-based programs. Longitudinal studies to evaluate this transition are a future research priority.

Despite most veterans’ positive experiences in the group exercise sessions, some veterans had a neutral or negative group experience. Machielse [41] developed 8 typologies of socially isolated individuals, which may help identify individuals who would readily benefit from group sessions (eg, the “Actives”) and those individuals who could benefit from group sessions but may need additional support to meaningfully impact their social health (eg, the “Outsiders” or the “Dependents”). Finally, we also need to consider that group sessions may be detrimental to some individuals’ social health and that the presence of group sessions in the program may be a deterrent for some veterans. These considerations can help us further tailor the program in the future, particularly among veterans who experience difficulties in group situations.

In addition to group experiences, some veterans discussed how the program impacted their interpersonal relationships external to the program. The veterans who spoke about their social relationships noted that the physical benefits of the program enhanced their ability to participate in activities they valued with their family or friends and made them less dependent on others for assistance with daily activities and household chores. Furthermore, at least 2 veterans discussed how either their spouse or family member was central to the plans they made to continue exercising following the program. Not all key informants described changes in social health and interpersonal relationships outside of the program. Such variable experiences are likely contingent to some degree upon existing social networks and interpersonal dynamics that may be beyond the purview of the program’s influence.

Component 4, technology, was used to address contextual factors (biopsychosocial) in which this program occurred and barriers and facilitators to behavior change (SCT). Generally, the technology facilitated program engagement, primarily through synchronous video visits. The Fitbit and Annie facilitated behavior change for some participants by supporting monitoring and accountability. Experiences using the Fitbit were similar to those reported by Andersen et al [42], who studied a cohort of patients with chronic heart disease. The barriers and facilitators that we identified—low competency, dissatisfaction, and assistance from family members—are consistent with prior research in older adults [43,44]. Importantly, findings from key informants influenced program adaptations, as detailed below in *Program Adaptations* section.

Veterans participated in the program during the early months of the COVID-19 pandemic. Efforts taken to reduce viral spread resulted in canceled facility-based appointments and longer wait times for physical therapy. During the early stages of the pandemic, the VA implemented a triage system in which only high-priority patients were seen in person, including those with conditions that, if left untreated by physical therapy, would have lasting detrimental health effects. This environment increased the demand for and acceptance of the telehealth program, especially among older veterans with multiple chronic conditions and compromised immune systems. Some participants voiced that these concerns served as an impetus for participating in the telehealth program. Because of this unique context (ie, a global pandemic), it is unclear whether veterans will continue to view this program as feasible and acceptable after the pandemic. Finally, rurality can be a significant barrier within the VA system to receiving in-person care and is often a reason cited to support the expansion of telehealth services for a variety of specialties [45-47]. Of the 15 participants in our cohort, 4 (27%) were rural, and for most of them, the telehealth program was the only feasible option for receiving physical therapy services within the VA health care system.

### Program Adaptations

Key informants provided detailed feedback regarding the program components, enabling us to identify opportunities for improvement. Primary program adaptations were identified for the biobehavioral intervention (coaching), group sessions (transition from individual to group sessions and group session dynamics), and technology supports. The coaching sessions were not as acceptable to veterans as compared with the more traditional components of the physical therapy program for a variety of reasons, including the increased time commitment and lack of perceived value. The program staff also recognized that separating the coaching sessions resulted in a greater workload. Thus, we opted to integrate the coaching sessions into the individual physical therapy sessions for future iterations of the program. Findings from this program evaluation also highlighted the need to educate participants about the coaching component to enhance engagement.

Some participants perceived a lack of communication among the clinicians when transitioning from individual sessions to group sessions. Veterans in the latter half of the program often had a different physical therapist for their group sessions. Although our team had processes in place for team communication, this feedback prompted us to revisit these processes. We conducted weekly patient round meetings to ensure that the group therapist had all the information necessary for a successful transition. For the current iteration of the program, the group lead therapist joins an individual session before the first group session, when possible, to meet the veteran and observe how they exercise. This adaptation has been helpful for both veterans and the clinicians. Some difficulties experienced transitioning to the group sessions were a result of deviation from the telehealth protocol, and as a result, we developed a fidelity checklist to serve as both an assessment of intervention fidelity and an opportunity for ongoing staff training and feedback. Furthermore, within the group sessions, some veterans did not feel comfortable sharing health-related information, which was sometimes necessary to supplement information obtained privately during pre- and postvisit calls. To address this concern in the current program iteration, we work with each veteran to develop a communication plan in the event that they need to leave the session suddenly for a nonmedical emergency or they need to speak privately with staff during the session.

For the technology, we received feedback related to both the Annie text messaging protocol and the Fitbit activity monitors. Although most veterans enjoyed the text messages from Annie, some veterans did not want to receive certain messages or preferred to receive messages less often. On the basis of this feedback, we added more flexibility to the program’s protocol to allow for adjustments to the text messages, as needed. The veterans who enjoyed engaging with Annie also noted that they wished there was a way to respond to some of the 1-way text messages, typically those pertaining to goals. Because of this feedback, we updated the protocol to include 2-way text message templates that ask about goals. On Mondays, veterans were asked if they had set a weekly goal (yes or no response), and on Fridays, veterans were asked about their progress toward their goal (0-10 scale response). Regarding the activity monitor, most individuals used the Fitbit to monitor their daily step count; although this was sufficient for most veterans, other veterans felt that there were missed opportunities to use additional features of the activity monitor, such as those that help monitor sleep hygiene or track exercise intensity, and they perceived that the program staff were unaware of these additional data. As a result, we purposefully integrated Fitbit training into staff training, and we added veteran Fitbit training to the biobehavioral intervention protocol.

### Limitations

This was a program evaluation, and as such, the results of this evaluation are not generalizable beyond this specific program. Although the findings may be helpful for others who would like to start a similar program, local adaptations may be needed for the program to be successful in different contexts. The qualitative data provided information about different program components; however, the outcomes associated with some aspects of the program remain unclear. For example, we heard limited information about how the veterans perceived the group sessions to impact their social health, if at all. Although veterans participated in up to 24 group sessions, not all veterans were able to experience a robust group because of program challenges that impacted our ability to maintain a steady census. As a result, some veterans completed group sessions during which they were the only individual in the group. We also did not interview participants who withdrew before 8 weeks of the program, and it is possible that we did not capture some of the barriers associated with program participation. Future program evaluations and research studies should include such individuals to understand their experiences. Interviews were also conducted immediately after the end of the program; therefore, we do not know how the program may contribute to longer-term outcomes. Furthermore, we had limited information about what behavior change skills veterans perceived to be the most helpful and how those skills would continue to be used in the future.

### Conclusions

The findings from this qualitative program evaluation supported ongoing quality and process improvements. The key facilitators for program engagement that emerged were the development of both a trusting therapeutic relationship and peer comradery, the presence of a strong social network of family and friends, and the convenience and efficiency associated with accessing telehealth physical therapy services. Technology served as a facilitator for those who identified value in using the digital tools but was a barrier for those who were dissatisfied with the tool. The program logistics of the group sessions were a barrier for participants who were working, and there was a subset of key informants who did not find value in the coaching sessions. Both findings led to improvements in the program. The findings from this evaluation may be adapted for the development of similar programs in different contexts. Further research is needed to develop a deeper understanding of how program components influence social health and longer-term behavior change.

## Appendix

Multimedia Appendix 1 Details of the multicomponent telehealth program.

Multimedia Appendix 2 Semistructured interview guide.

## Abbreviations

MoCA: Montreal Cognitive Assessment
SCT: social cognitive theory
VA: Veterans Administration

## Data Availability

The participants of this program evaluation did not provide written consent for their data to be shared publicly, and the authors confirm that the data supporting the findings of this study are available in the manuscript.
