# Supplementary material for: Older Veterans’ Experiences of a Multicomponent Telehealth Program: Qualitative Program Evaluation Study
Source: JMIR Form Res. 2023 Sep 8;7:e46081. doi: 10.2196/46081 (PMC10517383; doi:10.2196/46081)
Supplement: Multimedia Appendix 1 [file formative_v7i1e46081_app1.docx]

**Appendix 1** Details of the Multicomponent Telehealth Program

## Overview of Multicomponent Telehealth Program

The 12-week multicomponent program (Table 1) was delivered by licensed physical therapists and a physical therapy assistant. There were 4 core components to the program: 1) high-intensity rehabilitation [19] delivered during hour-long individual and group physical therapy sessions; 2) biobehavioral interventions [20, 23] to support physical activity behavior change and program participation; 3) social support via group physical therapy; and 4) technology to augment intervention delivery and enhance participation.

| **Table 1.** Timeline of Telehealth Program Components | | | | | | | | | | | | |
| --- | --- | --- | --- | --- | --- | --- | --- | --- | --- | --- | --- | --- |
|  | **Program Week** | | | | | | | | | | | |
| **Component** | **1** | **2** | **3** | **4** | **5** | **6** | **7** | **8** | **9** | **10** | **11** | **12** |
| **1) High-Intensity Rehabilitation**  Individual PT (10 to 12 sessions) | 2 | 2 | 2 | 2 | 1 | 1 |  |  |  |  |  |  |
| **2) Biobehavioral Intervention**  (8 sessions) | 1 | 1 | 1 | 1 | 1 | 1 |  | 1 |  |  | 1 |  |
| **3) Social Support**  Group PT (20 to 24 sessions) |  |  | 1 | 1 | 2 | 2 | 3 | 3 | 3 | 3 | 3 | 3 |
| **4) Technology** | ✓ | ✓ | ✓ | ✓ | ✓ | ✓ | ✓ | ✓ | ✓ | ✓ | ✓ | ✓ |
| **Abbreviations:** PT: physical therapy | | | | | | | | | | | | |

### Component 1: High-Intensity Rehabilitation

High-intensity rehabilitation interventions were employed during both individual and group physical therapy sessions. Veterans started the program completing individual sessions (ranging from 10 to 12 sessions) and then transitioned into group sessions (ranging from 20 to 24 sessions). High-intensity rehabilitation was defined as 80% of a 1-repetition maximum (8-RM) [24], and patients were instructed how to achieve this dosing during strengthening exercises (e.g., hip abduction) and functional movements (e.g., step-ups, sit to stands, floor transfers). Use of this high-intensity approach does not require testing of a 1-repetition maximum, but rather uses the principle of 8 repetitions to technical failure. Physical therapists were trained on identifying various signs of technical failure such as form deterioration, use of compensatory movement patterns, and loss of eccentric control. If a participant was able to perform an exercise for 5 to 8 repetitions with technical failure by the sixth to ninth repetition, then we considered high-intensity dosing to be achieved. If a participant performed less than 5 repetitions, the exercise was too challenging (dose was ≥ 90% of 1-RM), and if a participant could perform 9 or more repetitions without signs of technical failure, the exercise was too easy (dose was < 80% of 1-RM); in both scenarios, the therapist would modify accordingly to help the participant achieve the intended dosing (Table 2).

| **Table 2.** Application of high-intensity rehabilitation principle | | |
| --- | --- | --- |
| **Repetitions Completed** | **Dose**  **(% of 1-RM)** | **Adjustment for next set or next session** |
| 0 to 4 | ≥ 90% | Decrease resistance or modify to an easier exercise that targets the same muscle groups |
| 5 to 8 | 80% | Maintain resistance of exercise |
| 9 or more | < 80% | Increase resistance or modify to more challenging exercise that targets the same muscle groups |

### Component 2: Biobehavioral Interventions

A different physical therapist trained in motivational interviewing techniques [25] delivered eight 30-minute biobehavioral intervention sessions to facilitate program engagement and physical activity behavior change. These sessions were used to help Veterans develop skills such as self-monitoring, tailored feedback, identifying barriers and facilitators, and action planning (e.g., goal development). Data from the Fitbit (or other activity monitor) was integrated throughout these various skills and was mostly relevant for self-monitoring and action planning. For example, most Veterans set goals related to daily step counts, and thus, used the Fitbit to track their daily activity (self-monitoring) and adjust weekly goals (action planning).

Physical therapists used motivational interviewing techniques [25] to deliver these biobehavioral interventions. Motivational interviewing is founded on collaboration, evocation, and autonomy and is designed to elicit an individual’s intrinsic motivations for change, which subsequently served as the focus of coaching support. Motivational interviewing uses techniques such as open-ended questions and affirmations to establish the individual’s readiness and priorities for behavior change in order to focus on what matters most to each individual [25].

### Component 3: Social Support

Social support was integrated into the group PT sessions and facilitated by the physical therapists and group assistants to allow Veterans to share their experiences, successes, and struggles during the program with the goal of addressing loneliness. During group physical therapy sessions, clinicians and staff purposefully engaged Veterans by providing encouragement on performance and facilitated peer support. Each session also included an “ice breaker” question at the beginning of the session to allow Veterans to share information about themselves and get to know their fellow Veterans in a low-risk manner. To help foster an inclusive and supportive environment, all Veterans received a copy of the group telehealth agreement and consent document developed by the Puget Sound VA; this document outlined expectations for group settings including maintaining confidentiality of the group and treating others with respect. The agreement also explicitly stated that violent or intimidating behavior would not be tolerated.

### Component 4: Technology

Technology supports included a videoconference platform (VA Video Connect), text messaging platform (Annie), activity monitor (Fitbit), and data sharing application (Sync My Health Data). Most program related sessions occurred synchronously using VA Video Connect; occasionally, coaching sessions occurred over telephone when the Veteran was unable to join the VA Video Connect session. The Annie text messaging protocol was developed specifically to support the telehealth program by delivering both one-way and two-way text messages. One-way text messages were educational, encouraging, and/or prompts for group discussions; these messages did not request a response from the individual. Two-way text messages were used to collect data from individuals and included 1) daily step counts and 2) fall occurrence (once weekly). The Fitbit was purposively integrated into the coaching sessions described above and served as the primary means for self-monitoring physical activity. The data sharing application, Sync My Health Data, allowed Veterans to share their Fitbit data directly with their VA physical therapists. While this activity data was primarily used by the Veteran’s coach to facilitate behavior change sessions, the individual and/or group physical therapist could also access the data, providing support for behavior change across all sessions.
