# Supplementary material for: Older Veterans’ Experiences of a Multicomponent Telehealth Program: Qualitative Program Evaluation Study
Source: JMIR Form Res. 2023 Sep 8;7:e46081. doi: 10.2196/46081 (PMC10517383; doi:10.2196/46081)
Supplement: Multimedia Appendix 2 [file formative_v7i1e46081_app2.docx]

**Appendix 2.** Semi-structured Interview Guide

## General/Opening Questions

1. What was it like to participate in an exercise program remotely? Briefly, what was the best thing and the most difficult thing about the program?
2. What made you want to participate in this program?

## Technology

Fitbit (or other activity monitor)

1. How did you use your Fitbit?
2. What did you do with the information from your Fitbit?
3. What was it like for you to learn how to use the Fitbit and accompanying app on your phone?
4. How do you feel the Fitbit affected your participation in the program?

Annie (Text Messaging)

1. Tell me about your experience with the Annie text messages. What did you do with the information from Annie?
2. What is the one thing you liked best about Annie?
3. What did you dislike about Annie?
4. What suggestions do you have to improve Annie?

## Logistics of the Program – duration, frequency, length of sessions

1. How did you feel about the duration of the program? (12 weeks)
2. How did you feel about the number of days per week you were asked to participate in the program? (3 to 4 days)
3. How did you feel about the length of the sessions? (Usually one hour)

## Group Sessions

1. What was the experience of exercising in a group but virtually like for you?
   1. *If key informant had experience exercising in in-person groups:* How did this feel similar to your previous experience? How was this different?
2. How did you feel in the group setting?
3. How prepared did you feel to start exercising in a group setting?

## Safety

1. Was there ever a time when you felt uncomfortable about doing a particular activity because you did not feel it was safe for you to do?

**If yes:** Could you tell me more about that experience or circumstances?

**If no:** What was it about the program that made you feel safe?

## Coaching Sessions

1. What did you think of the coaching sessions overall?
2. How did the coaching sessions affect your exercise/physical activities during the program?
3. Is there anything you took away from the coaching sessions that you have found you continue to use or do? Please share any examples.
4. What, ideally, would you want to get out of coaching sessions?

## Previous or New Perceptions of Exercise & Physical Activity; Program Outcomes

1. How did you feel about exercise before starting the program?
2. How is that feeling about exercise different or the same now?
3. Tell me about some of the challenges you experienced during the program. How did you overcome these obstacles?
4. What was the most meaningful change you saw in yourself?
5. Think back on what a typical day looked like for you prior to this program and think about what your typical day looks like now. What is different? What is the same?

## Closing question

1. What else about the program and your experience would you like to share?
